# Correlations Study Between ^18^F-FDG PET/CT Metabolic Parameters Predicting Epidermal Growth Factor Receptor Mutation Status and Prognosis in Lung Adenocarcinoma

**DOI:** 10.3389/fonc.2019.00589

**Published:** 2019-07-18

**Authors:** Bin Yang, Qing gen Wang, Mengjie Lu, Yingqian Ge, Yu jun Zheng, Hong Zhu, Guangming Lu

**Affiliations:** ^1^Department of Medical Imaging, Jinling Hospital, Medical School of Nanjing University, Nanjing, China; ^2^Department of Medical Imaging, Jinling Hospital, Clinical School of Southern Medical University, Nanjing, China; ^3^Department of Medical Imaging, Jinling Hospital, Nanjing, China; ^4^Siemens Healthineers Ltd., Shanghai, China; ^5^Department of Nuclear Medicine, Jinling Hospital, Medical School of Nanjing University, Nanjing, China

**Keywords:** lung adenocarcinoma, PET/CT, metabolic parameters, EGFR, prognosis

## Abstract

**Purpose:** This study assessed the ability of metabolic parameters from ^18^Fluorodeoxyglucose positron emission tomography/computed tomography (^18^F-FDG PET/CT) and clinicopathological data to predict epidermal growth factor receptor (EGFR) expression/mutation status in patients with lung adenocarcinoma and to develop a prognostic model based on differences in *EGFR* expression status, to enable individualized targeted molecular therapy.

**Patients and Methods:** Metabolic parameters and clinicopathological data from 200 patients diagnosed with lung adenocarcinoma between July 2009 and November 2016, who underwent ^18^F-FDG PET/CT and *EGFR* mutation testing, were retrospectively evaluated. Multivariate logistic regression was applied to significant variables to establish a prediction model for *EGFR* mutation status. Overall survival for both mutant and wild-type *EGFR* was analyzed to establish a multifactor Cox regression model.

**Results:** Of the 200 patients, 115 (58%) exhibited *EGFR* mutations and 85 (42%) were wild-type. Among selected metabolic parameters, metabolic tumor volume (MTV) demonstrated a significant difference between wild-type and mutant *EGFR* mutation status, with an area under the receiver operating characteristic curve (AUC) of 0.60, which increased to 0.70 after clinical data (smoking status) were combined. Survival analysis of wild-type and mutant *EGFR* yielded mean survival times of 34.451 (95% CI 28.654–40.249) and 53.714 (95% CI 44.331–63.098) months, respectively. Multivariate Cox regression revealed that mutation type, tumor stage, and thyroid transcription factor-1 (TTF-1) expression status were the main factors influencing patient prognosis. The hazard ratio for mutant *EGFR* was 0.511 (95% CI 0.303–0.862) times that of wild-type, and the risk of death was lower for mutant *EGFR* than for wild-type. The risk of death was lower in TTF-1-positive than in TTF-1-negative patients.

**Conclusion:** ^18^F-FDG PET/CT metabolic parameters combined with clinicopathological data demonstrated moderate diagnostic efficacy in predicting *EGFR* mutation status and were associated with prognosis in mutant and wild-type *EGFR* non-small-cell lung cancer (NSCLC), thus providing a reference for individualized targeted molecular therapy.

## Introduction

Lung cancer is one of the most aggressive malignant tumors, with high rates of morbidity and mortality worldwide, and has recently risen to rank first among malignant tumors (1–3). Among all pathological categories of lung cancer, non-small cell lung cancer (NSCLC) is the most common, accounting for ~85% of lung cancers (4–6). Recent advances in the understanding of the molecular biology of NSCLC (7, 8) have attracted attention for molecular-driven targeted therapy. Tyrosine kinase inhibitors (TKIs), such as gefitinib and erlotinib, have been introduced (9) and have become an effective treatment for patients with mutations in the epidermal growth factor (*EGFR*) gene (10, 11). Several studies (12–14) have described that the *EGFR* mutation status is the main factor in predicting the therapeutic effect of EGFR- TKIs. Therefore, it is essential to identify *EGFR* mutation status before attempts at targeted therapy.

However, tumor biopsies for the detection of *EGFR* mutations have limitations. The tumor biopsy location may strongly affect the detection result, and patients' general condition may also restrict widespread use of biopsies in clinical practice. Therefore, the use of medical imaging as a non-invasive method to obtain information about the tumor phenotype could provide clues to predict mutation status of the *EGFR* gene and has been investigated in several studies (15–17) using positron emission tomography with 2-deoxy-2-[fluorine-18]fluoro-D-glucose integrated with computed tomography (^18^F-FDG PET/CT) to predict the mutation status of *EGFR* in patients with lung adenocarcinoma; however, the results remain controversial.

Additionally, studies have also described the effects of targeted therapy associated with *EGFR* mutation status. However, few studies have investigated the effect of various metabolic parameters and clinicopathological information on the prognosis of patients with wild-type and mutant type *EGFR*. Therefore, the objectives of the present study were to assess the ability of metabolic parameters from ^18^F-FDG PET/CT and clinicopathological data to predict epidermal growth factor receptor (EGFR) expression/mutation status in patients with lung adenocarcinoma, and to develop a prognostic model based on differences in *EGFR* mutant status, thereby providing a reference for individualized molecular targeted therapy.

## Methods

### Patients

Clinicopathological data from 200 patients diagnosed with lung adenocarcinoma between July 2009 and November 2016, were retrospectively analyzed. Patients who underwent PET/CT examination before treatment with histopathological confirmation, along with *EGFR* gene test results were included. Individuals with a history of malignant tumors, previous treatment before *EGFR* gene testing, and insufficient tissue for genetic testing were excluded from this study. Clinicopathological data included age, sex, family history, smoking history/status (Non-smokers were defined as patients who never smoked or smoked <100 cigarettes in their lifetimes), histological grade, regional lymph node metastasis, distant metastasis, tumor, node, and metastasis stage (i.e., I, II/III, and IV), thyroid transcription factor-1 (TTF-1) (– or one + was defined as negative; ≥2 was defined as positive), Ki-67 ( ≤ 25% was defined as low expression and >25% as high expression), carcinoembryonic antigen, tumor location, the largest diameter of the tumor, and PET/CT metabolism parameters are summarized in Table 1. Overall survival (OS) defined as the time interval between date of PET/CT examination and death or to the date of final follow-up. Statisticed by month. OS of patients with mutant and wild-type *EGFR* was followed up (July 2009 to January 2019). The starting point for OS was the date of PET/CT examination and the end point was defined as the date of telephonic follow-up or death. And the telephonic follow-up was the last follow-up.

**Table 1:** Association between clinical characteristics and the epidermal growth factor receptor mutation status in patients with lung adenocarcinoma.

**Characteristic**	**Wild-type**	**Mutant-type**	***P*-value**
**Age, mean** **±** **SD, years**	63.61 ± 11.42	59.39 ± 9.73	0.005
**Gender, No. (%)**			0.001
Male	58 (68.2)	50 (43.5)	
Female	27 (31.8)	65 (56.5)	
**Family history, No. (%)**			0.870
No	81 (95.3)	109 (94.8)	
Yes	4 (4.7)	6 (5.2)	
**Smoking status, No. (%)**			<0.001
Never	43 (50.6)	89 (77.4)	
Ever	42 (49.4)	26 (22.6)	
**Histologic grade, No. (%)**			0.015
Poorly differentiated	27 (41.5)	20 (27.8)	
Moderately differentiated	33 (50.8)	36 (50.0)	
Well-differentiated	5 (7.7)	16 (22.2)	
**Lymph node metastasis, No. (%)**			0.422
No	26 (30.6)	29 (25.4)	
Yes	59 (69.4)	85 (74.6)	
**Distant metastasis, No. (%)**			0.160
No	30 (35.3)	30 (26.1)	
Yes	55 (64.7)	85 (73.9)	
**Stage, No. (%)**			0.093
I/II	15 (17.6)	11 (9.6)	
III/IV	70 (82.4)	104 (90.4)	
**TTF-1, No. (%)**			0.021
–	9 (15.8)	4 (4.5)	
+	48 (84.2)	84 (95.5)	
Ki-67, median [P_25_–P_75_]	30 [20~50]	30 [10~40]	0.178
CEA, median [P_25_–P_75_]	4.70 [2.95~27.25]	11.80 [2.40~61.90]	0.270
Diameter, median [P_25_–P_75_], mm	34.00 [23.00~48.00]	33.00 [23.00~46.00]	0.396
**Site, No. (%)**			0.036
Right upper lobe	19 (22.4)	38 (33.3)	
Right middle lobe	3 (3.5)	8 (7.0)	
Right lower lobe	15 (17.6)	23 (20.2)	
Left upper lobe	19 (22.4)	27 (23.7)	
Left lower lobe	29 (34.1)	18 (15.8)	

*TTF-1, thyroid transcription factor- 1; CEA, carcinoembryonic antigen*.

### PET/CT Imaging Method and Image Acquisition

All patients underwent PET/CT before initiation of treatment. PET/CT scans were performed using a PET/CT imager(Biograph 16, Siemens, Knoxville, TN, USA).^18^F-FDG was produced by EBCO's TR19 medical cyclotron in Canada, and then automatically synthesized using a chemical synthesis system with a radiochemical purity >95%. All patients fasted 6–8 h before the examination. Height, weight, and fasting blood glucose levels were recorded before the examination and blood glucose levels were controlled to levels <6.7 mmol/L. After an intravenous injection of ^18^F-FDG (3.7–6.66 MBq/kg), patients were asked to consume 500–1,000 ml water and rest for 40–60 min. A whole-body PET/CT examination was performed from the skull base to the upper femur (Generally 6–7 beds location). Both CT and PET scan were performed. CT scan parameters were as follows: tube voltage, 120 kV; tube current, 140 mAs; and layer thickness and layer spacing, 5 mm. The PET acquisition method involved a three-dimensional acquisition, 3 min/bed, and the image was reconstructed by iterative reconstruction. Images of transverse, sagittal, and coronal positions of CT, PET, and PET/CT fusions were acquired.

### Image Analysis and Measurement of Parameter Values

Two experienced nuclear medicine physicians marked the location, size, and shape of the tumor and its relationship with surrounding tissues on post-processed images. When the opinions of the two physicians are inconsistent, it is decided by the collective discussion of our department. The imaging data were transmitted to the AW4.6 workstation and tumor lesions were identified by visual inspection. Semi-quantitative measurements were based on the high FDG metabolic area of the lesion using the MS viewer software and by manually delineating the region of interest (ROI). ROIs were placed over the primary tumor to measure the maximum standard uptake value (SUVmax) (SUVmax threshold is set to 40%), mean standard uptake value (SUVmean).Calculate the metabolic tumor volume (MTV) (ROI area per layer × layer thickness = volume of each layer, then add the volume of each layer to get MTV). And then calculate tumor-lesion glycolysis (TLG) (TLG = SUVmean × MTV).

### *EGFR* Gene Detection

*EGFR* gene testing was performed by the pathology department at the authors' hospital. The tissues for gene detection were obtained from a specimen of tumor resection or biopsy, fixed in formalin, embedded in paraffin, and cut into sections. *EGFR* gene detection was performed using a real-time fluorescent quantitative nucleotide amplification method.

### Statistical Analysis

All data were processed using SPSS version 25.0 (IBM Corporation, Armonk, NY, USA). Quantitative data that were normally distributed are expressed as mean ± standard deviation, and the independent sample *t*-test was used for comparison between the two groups. Quantitative data that were not normally distributed are expressed as median (interquartile range), and the Mann-Whitney test was used for comparison between the two groups. Qualitative data are expressed as number and percentage (n [%]), and the chi-squared test or Fisher's exact probability method was used for comparison between the two groups. SUVmax, SUVmean, TLG, MTV index, and area under the receiver operating characteristic (ROC) curve (AUC) were calculated from the ROC curve, the identification of wild-type and mutant *EGFR* status at any threshold, and the maximum Youden's index as the standard for selecting the optimal cut-off limit value to convert the four quantitative indicators into two-category indicators. Covariates were screened using univariate logistic regression (*p* < 0.10 factor), and further forward likelihood ratio (LR) was used (inclusion test level = 0.05, rejection test level = 0.10) to establish a multivariate logistic step-wise regression model of predictive factors. Multivariate analysis was performed to construct the model; the odds ratio (OR), 95% confidence interval (CI), and AUC were calculated; an optimal cut-off point was identified using Youden's index; the 95% CI values of the cut-off point were calculated. The Delong method was used to compare the AUC values of the different models.

Survival rates for *EGFR* wild-type and mutant patients were analyzed using the Kaplan-Meier method. Survival time is expressed as mean and median and 95% CI, and compared using the log-rank test. A Cox proportional hazard regression model was used to screen covariates, including variables with *p* < 0.10 in the univariate analysis, and further forward LR (incorporated with a test level of 0.05 and a rejection test level of 0.10). The optimal multivariate Cox regression model was established and the hazard ratio (HR) and corresponding 95% CI were calculated; *p* < 0.05 was considered to be statistically significant.

## Results

### Patient Information

The clinical characteristics of the patient population are summarized in Table 1. Of the 200 patients, 115 (58%) exhibited an *EGFR* mutation and 85 (42%) exhibited wild-type *EGFR*. The mean age of the 200 patients with *EGFR* wild-type and mutant lung adenocarcinoma was 63.61 ± 11.42 and 59.39 ± 9.73 years, respectively. The average age of the *EGFR* wild-type patients was greater than those with mutant *EGFR*. Among the 200 patients, 108 (54%) were male, 92 (46%) were female, 132 (66%) were non-smokers, and 68 (32%) were smokers. The *EGFR* mutation status of poorly differentiated, moderately differentiated, and highly differentiated tumors was 47 (28.1%), 99 (59.3%), and 21 (12.6%), respectively. TNM stages I and II, and III and IV, accounted for 26 (13%) and 174 (87%), respectively. A total of 200 patients were followed-up from July 2009 to January 2019; eight (4%) patients were lost to follow-up. Of the 192 patients who were followed-up, the median survival time was 36.000 months [95% CI 31.284–40.716 (range 1–114 months)] and the mean survival time was 47.635 months (95% CI 40.610–54.661). The 1-, 3-, and the 5-year OS rates were 84.9, 47.7, and 30.5%, respectively. In the follow-up of 123 (64.1%) patients, the median survival time was 21.000 months (range 1–83 months) (95% CI 16.245–25.755), and mean survival time was 24.472 months (95% CI 21.837–27.107). The 1-, 3-, and 5-year OS rates were 76.4, 19.5, and 2.4%, respectively.

### The Relationship Between Clinical Features and *EGFR* Mutations

*EGFR* mutations were more common in patients who were female (56.5 vs. 43.5%; *p* < 0.001), non-smokers (77.4 vs. 22.6%; *p* < 0.001), patients with moderately differentiated tumors (27.8 vs. 50.0%, and 22.2%; *p* < 0.015), patients with TTF-1 positive expression (95.5 vs. 4.5%; *p* < 0.021), and patients with lung adenocarcinoma of the right upper lobe (Table 1).

### Relationship Between Metabolic Parameters and *EGFR* Mutation Status

**Figure 1:**
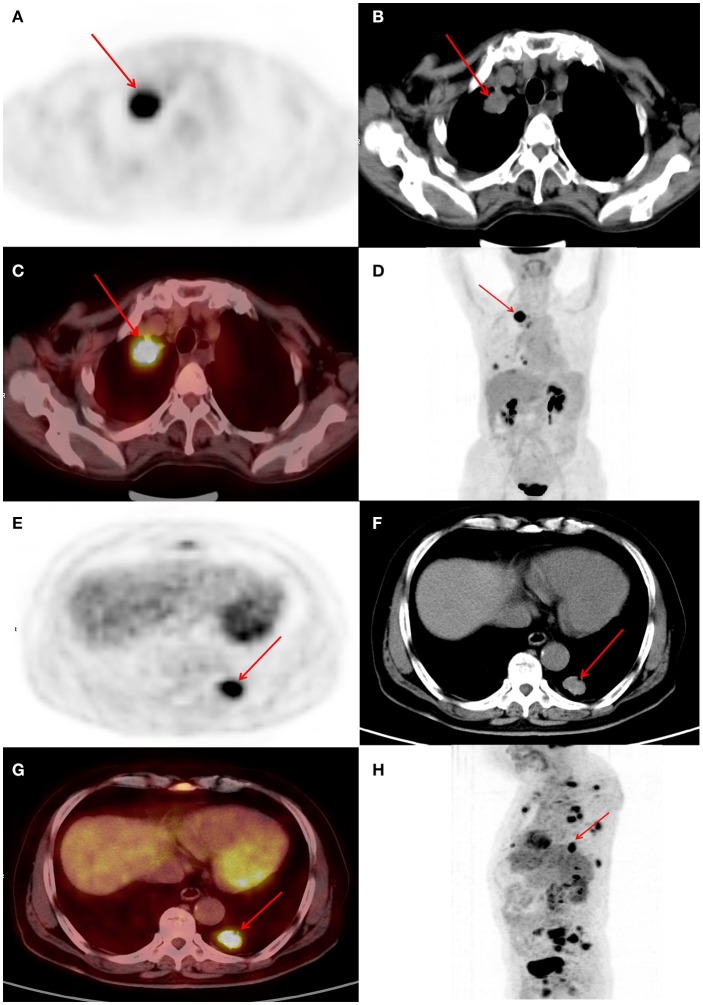
**(A–D)** Female, 63 years of age. Positron emission tomography/computed tomography (PET/CT) revealing lung adenocarcinoma of the right upper lobe, ~33 × 25 mm, increased fluorodeoxyglucose (FDG) metabolism, maximum standardized uptake value (SUVmax) 6.8, epidermal growth factor (*EGFR*) mutation, and overall survival (OS) of 29 months. **(E–H)** Male, 72 years of age. Positron emission tomography/computed tomography (PET/CT) revealing lung adenocarcinoma of the posterior basal segment of the left lower lobe, ~27 × 19 mm, increased fluorodeoxyglucose (FDG) metabolism, maximum standardized uptake value (SUVmax) 9.4, epidermal growth factor receptor (*EGFR*) mutation, and OS of 13 months.

ROC curves were used to classify a variety of continuous variables that predict the status of *EGFR*, and the optimal cut-off points were determined (Table 2). Representative PET/CT images of two patients with *EGFR* mutations are shown in Figure 1.

**Table 2:** Association between metabolic parameters and epidermal growth factor receptor mutation status.

**Characteristic**	**Wild-type**	**Mutant-type**	***P*-value**
**SUVmax, mean** **±** **SD**	8.43 ± 4.50	7.81 ± 4.18	0.310
**SUVmax, No. (%)**			0.012
>6.15	64 (75.3)	67 (58.3)	
≤ 6.15	21 (24.7)	48 (41.7)	
**SUVmean, mean** **±** **SD**	5.16 ± 2.73	4.89 ± 2.73	0.486
**SUVmean, No. (%)**			0.010
>3.53	67 (78.8)	71 (61.7)	
≤ 3.53	18 (21.2)	44 (38.3)	
**TLG (g), median [P_25_~P_75_]**	60.36 [19.99~153.24]	34.62 [17.37~77.14]	0.046
**TLG (g), No. (%)**			0.006
>59.19	43 (50.6)	36 (31.3)	
≤ 59.19	42 (49.4)	79 (68.7)	
**MTV (cm^3^), median [P_25_~P_75_],**	13.24 [5.98~25.77]	8.31 [4.08~19.99]	0.025
**MTV (cm**^**3**^**), No. (%)**			0.003
>11.55	49 (57.6)	42 (36.5)	
≤ 11.55	36 (42.4)	73 (63.5)	

*SUVmax, maximal standard uptake value; SUVmean, mean standard uptake value; MTV, metabolic tumor volume; TLG, total lesion glycolysis*.

### Prediction of *EGFR* Mutation Status

**Figure 2:**
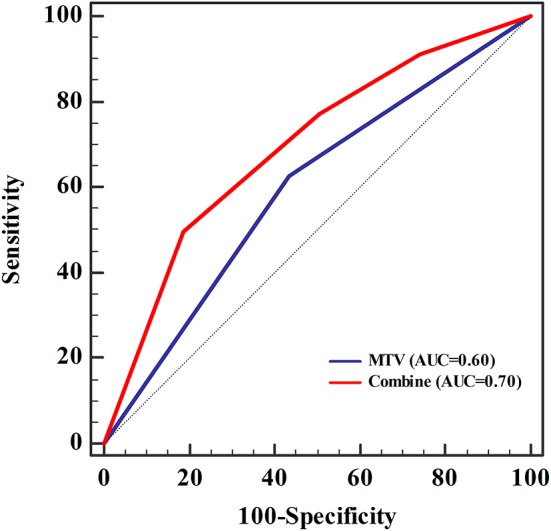
The area under the receiver operating characteristic curve (AUC) for metabolic tumor volume (MTV) was 0.60, which increased to 0.70 when clinical data (smoking status) was combined. Sensitivity and specificity were 0.50 and 0.81, respectively. The cut-off value of MTV was 11.55, and was 0.56 when combined with the clinical data (smoking status).

Univariate logistic regression analysis revealed a significant association between *EGFR* mutation status and age, sex, smoking status, histological grade, stage, TTF-1, SUVmax, SUVmean, TLG, and MTV. The multivariate model using forward LR stepwise regression analysis revealed that smoking and MTV remained as independent variables for predicting *EGFR* mutations [non-smoker (OR,0.288; *p* < 0.001); MTV (OR, 2.482; *p* = 0.003)]. Smoking and MTV were independent variables for constructing predictive models. MTV demonstrated a significant difference between wild-type and mutant *EGFR* status, with an AUC of 0.60, which reached 0.70 after clinical data (smoking status) was combined (Table 3 and Figure 2).

**Table 3:** Univariate and multivariate analysis of various predictive factors for the *EGFR* status in lung adenocarcinoma.

**Intercept and variable**	**Univariable logistic**			**Multivariable logistic**		
	****β****	**Odds ratio (95% CI)**	***P***	****β****	**Odds ratio (95% CI)**	***P***
Age	−0.039	0.961 (0.934~0.989)	0.006			
Gender (Female)	1.027	2.793 (1.553~5.022)	0.001			
Smoking status (Never)	−1.231	0.292 (0.159~0.538)	<0.001	−1.246	0.288 (0.154~0.539)	<0.001
Histologic grade			0.047			
Moderately differentiated	0.387	1.473 (0.698~3.107)				
Well-differentiated	1.463	4.320 (1.356~13.763)				
Stage (III/IV)	0.706	2.026 (0.879~4.669)	0.097			
TTF-1 (+/++/+++)	1.371	3.937 (1.151~13.471)	0.029			
Site, No. (%)			0.036			
Right upper lobe	19 (22.4)	38 (33.3)				
Right middle lobe	3 (3.5)	8 (7.0)				
Right lower lobe	15 (17.6)	23 (20.2)				
Left upper lobe	19 (22.4)	27 (23.7)				
Left lower lobe	29 (34.1)	18 (15.8)				
SUVmax ( ≤ 6.15)	0.781	2.183 (1.178~4.045)	0.013			
SUVmean ( ≤ 3.53)	0.836	2.307 (1.214~4.383)				
TLG (g) ( ≤ 59.19)	0.809	2.247 (1.258~4.012)	0.006			
MTV (cm^3^) ( ≤ 11.55)	0.861	2.366 (1.333~4.199)	0.003	0.909	2.482 (1.361~4.529)	0.003
Intercept				0.260		0.279
Sensitivity					0.50 (0.40~0.59)	
Specificity					0.81 (0.71~0.89)	

*TTF-1, thyroid transcription factor-1; SUVmax, maximal standard uptake value; SUVmean, mean standard uptake value; MTV, metabolic tumor volume; TLG, total lesion glycolysis*.

### Survival Analysis

**Figure 3:**
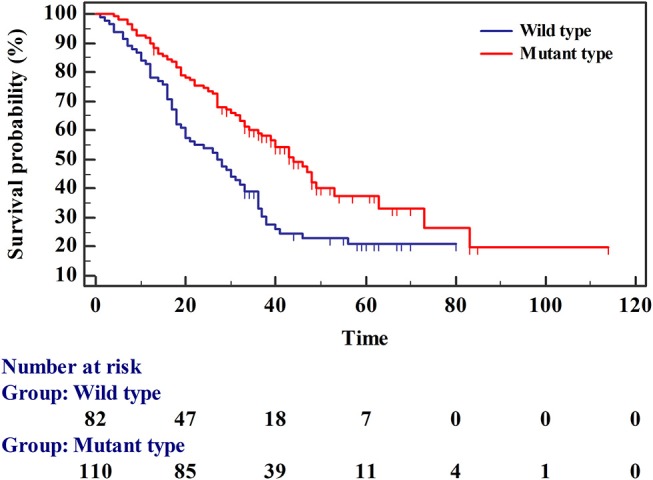
Survival curves for patients with wild-type and mutant epidermal growth factor receptor.

The survival rates of wild-type and mutant *EGFR* were analyzed using the Kaplan-Meier method. The average survival time of wild-type was 34.451 (95% CI 28.654–40.249), and the median survival time was 27 months (95% CI 20.000–33.000). The mean survival time of the mutant type was 53.714 (95% CI 44.331–63.098) and the median survival time was 44 months (95% CI 36.000–53.000) (Table 4 and Figure 3). The multivariate Cox regression model revealed mutation type, tumor stage, and expression status of TTF-1 as independent risk factors for patient prognosis. The HRs for TTF-1 (++/+++), stage (III/IV), and type (mutant) was 0.325 (95% CI 0.156–0.679; *p* = 0.003), 7.116 (95% CI 1.710–29.617; *p* = 0.007), and 0.511 (95% CI 0.303–0.862; *p* = 0.012), respectively (Table 5).

**Table 4:** Survival analysis for patients with wild-type and mutant patients epidermal growth factor receptor.

**Type**	**Mean**	**SE**	**95% CI for the mean**	**Median**	**95% CI for the median**
Wild type	34.451	2.958	28.654–40.249	27.000	20.000–33.000
Mutant type	53.714	4.787	44.331–63.098	44.000	36.000–53.000
Overall	47.857	3.600	40.801–54.912	36.000	30.000–40.000

**Table 5:** Establishment of the Cox proportional risk regression model.

**Variable**	**β**	**Hazard Ratio (95% CI)**	***P***
TTF-1 (++/+++)	−1.123	0.325 (0.156~0.679)	0.003
Stage (III/IV)	1.962	7.116 (1.710~29.617)	0.007
Type (Mutant)	−0.671	0.511 (0.303~0.862)	0.012

*TTF-1, thyroid transcription factor-1; HR, Hazard Ratio*.

## Discussion

In the present study, we investigated the association between PET/CT metabolic parameters and clinicopathological data and *EGFR* mutation status, and found a higher frequency of *EGFR* mutations in females and non-smokers. Previous epidemiological studies have also reported associations between specific features, such as female sex, non-smokers, and Asian origin with mutations in *EGFR* (18–22), consistent with our results. In addition, results of the present study demonstrated a relationship between *EGFR* mutation status and age and tumor stage, with the average age of wild-type patients greater than that of patients with mutant *EGFR*. *EGFR* mutation was primarily detected in stages III/IV, which has rarely been reported in previous studies. This could be due to race selection bias or insufficient sample size. Our study also found a significantly higher positive expression rate of TTF-1 in mutant patients, which may be explained by the high positive expression rate of TTF-1 in NSCLC and higher adenocarcinoma rate than squamous cell carcinoma, consistent with the literature (23, 24).The principle of applying PET/CT in lung cancer is based on differences in glucose metabolism between tumor and normal tissues (25). Studies have reported that the *EGFR* gene can affect GLUT-1 through a downstream pathway, thus further affecting tumor glucose metabolism. As such, differences in *EGFR* mutation status may lead to differences in tumor glucose metabolism (26).

In our study, univariate logistic regression analysis revealed a significant association between *EGFR* mutations and age, sex, smoking status, histological grade, stage, TTF-1, SUVmax, SUVmean, TLG, and MTV. Further multivariate analysis revealed MTV as a predictor of *EGFR* mutations, which was not reported in previous studies wherein SUVmax was used as a predictor for *EGFR* mutations (27–33). As an indicator reflecting the overall metabolic level of the tumor, MTV is suitable for predicting the mutation status of *EGFR*. In addition, studies have shown that patients with lower MTV have a better prognosis (34–36), while others have reported a significant association between patient prognosis and *EGFR* mutation status, thus supporting the results of our study. Our research demonstrated that the AUC of MTV was 0.60; further analysis using a combination of MTV with clinical data, including smoking history, resulted in an AUC of 0.70. A previous study involving 849 patients with NSCLC reported similar ^18^F-FDG PET/CT results to predict *EGFR* mutation status (32). The results revealed that the AUC was 0.557 when *EGFR* status was predicted using metabolic parameters alone. After combining clinical data, such as sex and smoking status, the AUC was 0.697. Mei et al. (37) used CT texture features to predict *EGFR* mutation status in 296 patients. The AUC predicted by histological features alone was 0.575, which increased to 0.664 after clinical data were combined. Zhao et al. (38) used clinical imaging features of CT to predict *EGFR* mutation status in 471 patients with lung adenocarcinoma and reported an AUC of 0.706 when *EGFR* mutation status was predicted by clinical features, and 0.784 after the combined CT signatures. In our study, the AUC for predicting *EGFR* mutation status was similar to that reported in previous studies.

Our study analyzed the survival rates of *EGFR* wild-type and mutant patients, revealing a shorter mean survival time for *EGFR* wild-type compared with that for the mutant (34.451 months [95% CI 28.654–40.249] and 53.714 months [95% CI 44.331–63.098], respectively). Median survival of the *EGFR* wild-type and mutant patients was 27 (95% CI 20.000–33.000) and 44 (95% CI 36.000–53.000) months, respectively. Our results demonstrated a better prognosis for patients with *EGFR* mutations than for those with EGFR wild-type. Multivariate Cox regression analysis revealed that mutation type, tumor stage, and expression status of TTF-1 were independent factors affecting patient prognosis. The HR for wild-type mutations was 0.511. The risk of death in the mutant type was 0.511 times that of the wild-type; specifically, the risk of death in the mutant type was lower than that of the wild-type, and the prognosis for the mutant type was better than that for the wild-type, which is consistent with previous results (10, 39, 40). The risk of death in stage III/IV was 7.116 times higher than that in stage I/II. The HR for TTF-1 positive expression was 0.325 times that for TTF-1 negative expression, indicating that the risk of death from positive expression was 0.325 times that from negative expression (i.e., the risk of death from positive expression was lower than that from negative expression), which is consistent with previous reports (41–43). The positive expression rate of TTF-1 in *EGFR* mutant patients was higher, indicating that the prognosis of patients with EGFR mutations was better than those with wild-type *EGFR*.

There were limitations to the present study, which primarily included its retrospective design, small sample size, and possible patient selection bias. In addition, owing to the small sample size, the exact site of the *EGFR* mutations was not analyzed. Furthermore, mutant-type and wild-type treatments were not analyzed; therefore, the impact of different treatments on prognosis could not be analyzed and, as such, further research is needed.

In summary, the MTV metabolic parameters of PET/CT had a certain value for the identification of mutant and wild-type *EGFR*. Combining clinicopathological data with metabolic parameters may further improve diagnostic efficiency and may evaluation prediction of the prognosis of patients with mutant and wild-type *EGFR*. Our results may provide a reference for the design and implementation of individualized molecular-targeted therapies.

## Data Availability

The raw data supporting the conclusions of this manuscript will be made available by the authors, without undue reservation, to any qualified researcher.

## Ethics Statement

The institutional review board of Jinling Hospital, Medical School of Nanjing University approved this retrospective study and waived the need to obtain informed consent from the patients.

## Consent for Publication

Patients consented to publishing their images and clinical information.

## Author Contributions

BY conceived the idea of the study and wrote the manuscript. BY, QW, and YZ collected the data. HZ and GL performed image analysis. ML performed the statistical analysis. YG and GL edited and reviewed the manuscript. All the authors discussed the results and commented on the manuscript.

### Conflict of Interest Statement

YG was employed by Siemens Healthineers Ltd. The remaining authors declare that the research was conducted in the absence of any commercial or financial relationships that could be construed as a potential conflict of interest.

## Abbreviations

CI: confidence interval
HR: hazard ratio
OR: odds ratio
PET/CT: positron emission tomography/computed tomography
MTV: metabolic tumor volume
NSCLC: non-small-cell lung cancer
EGFR: epidermal growth factor receptor
FDG: fluorodeoxyglucose
OS: overall survival
ROI: region of interest
SUVmax: maximum standardized uptake value
SUVmean: mean standardized uptake value
TLG: tumor lesion glycolysis
TKI: tyrosine kinase inhibitor
TTF-1: thyroid transcription factor-1.
